# Supplementary material for: Laquinimod Supports Remyelination in Non-Supportive Environments
Source: Cells. 2019 Oct 31;8(11):1363. doi: 10.3390/cells8111363 (PMC6912710; doi:10.3390/cells8111363)
Supplement: Supplementary file 1 [file cells-08-01363-s001.pdf]

cells

Laquinimod Supports Remyelination in Non-Supportive Environments

Stella Nyamoya [1,2,†], Julia Steinle [2,†], Uta Chrzanowski [3], Joel Kaye [4], Christoph Schmitz [3], Cordian Beyer [2] and Markus Kipp [2,5,*]

[1] Institute of Anatomy, Rostock University Medical Center, 18057 Rostock, Germany; Stella.Nyamoya@rwth-aachen.de
[2] Institute of Neuroanatomy and JARA-BRAIN, Faculty of Medicine, RWTH Aachen University, 52074 Aachen, Germany; Julia.Steinle@rwth-aachen.de (J.S.); CBeyer@ukaachen.de (C.B.)
[3] Department of Anatomy II, Ludwig-Maximilians-University of Munich, 80336 Munich, Germany; Uta.Chrzanowski@med.uni-muenchen.de (U.C.); Christoph_Schmitz@med.uni-muenchen.de (C.S.)
[4] AyalaPharma, VP Research & Nonclinical Development, Rehovot, 7670104, Israel; Joel Kaye joel.k@ayalapharma.com
[5] Centre for Transdisciplinary Neurosciences, Rostock University Medical Center, 18057 Rostock, Germany
* Correspondence: Markus.Kipp@med.uni-rostock.de; Tel.:+ 49 (0) 381 494 8401
† These authors contributed equally to this work

Supplementary Materials:

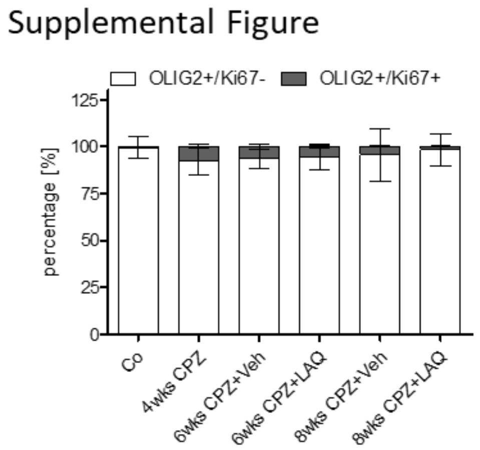

Figure S1: Percentage of proliferating (Ki67+) oligodendrocytes (OLIG2+) of control, showing results after 4 weeks of cuprizone intoxication, and 6 and 8 weeks of cuprizone intoxication for vehicle or laquinimod groups.
